# The Great Loop: From Conformal Cyclic Cosmology to Aeon Monism

**DOI:** 10.1007/s10838-024-09678-5

**Published:** 2024-09-24

**Authors:** Baptiste Le Bihan

**Affiliations:** Philosophy Department, University of Geneva, 2 Rue de Candolle, 1205 Geneva, Switzerland

**Keywords:** Conformal cyclic cosmology, Aeons, Time, Ontology, Metaphysics, Eternal return

## Abstract

Penrose’s conformal cyclic cosmology describes the cosmos as a collection of successive universes, the so-called aeons. The beginning and ending of our universe are directly connected to two other, anterior and posterior, universes. Penrose considers but rules out a different interpretation of conformal cyclic cosmology: that the beginning of our universe is connected to its own end in a cosmic loop. The paper argues that the view, aeon monism, should be regarded as a natural interpretation of conformal cyclic cosmology and discusses its implications for the concept of eternal return in light of the most popular metaphysics of time.

## Introduction

Did the universe have a beginning? This question can be empirically addressed, to some extent, by looking at various cosmological models either based on our best empirically confirmed theories in physics—*general relativity* and the *standard model of particle physics*—or on *quantum gravity*, a network of research programs in theoretical physics that aim at developing a novel, more explanatory, framework (Cao 2001). The second class of approaches finds examples in *eternal inflation* or *brane cosmology* (based on *string theory*) or *loop quantum cosmology* (a *loop quantum gravity* approach).^1^ Members of the first family of cosmological models either set aside the description of domains requiring a theory of quantum gravity—domains involving both quantum and gravitational aspects, where neither of these two phenomena can be ignored—or aim to find a way around to argue that even absent such a description, one can find evidence for a specific cosmological model in the empirically confirmed physics.

*Conformal cyclic cosmology*, elaborated by Roger Penrose, offers an example of the first family of views (Penrose 2006; 2010; 2014). The view is a fairly speculative alternative to the standard model of cosmology, the $\lambda$-CDM model. According to conformal cyclic cosmology, the natural world is an infinite succession of universes. The present paper offers a philosophical discussion of the ontological picture that underlies conformal cyclic cosmology. It investigates a particular step in Penrose’s reasoning, namely the move from the existence of antecedent and subsequent universes to the claim that those universes are other, numerically distinct universes. An alternative view is that these universes are in fact one and the same, the universe being closed in the timelike directions, just as it might be closed in the spacelike directions according to some cosmological models. Penrose considers briefly this view but rejects it. Call the view: *aeon monism*. According to aeon monism, not only has the universe never begun, but it will never end, for our past and our future are but one.

The paper argues, on the assumption that conformal cyclic cosmology is correct, that aeon monism should be considered a viable alternative to *aeon pluralism*—Penrose’s view that the universe is made of a plurality of aeons—and discusses some of its philosophical consequences for the philosophy of time. Since at this stage of research, it is still not known whether the sequence of successive aeons had a beginning (as in eternal inflation) or is past-infinite, the question of whether the universe—understood as the collection of aeons—had a beginning with a first aeon remains open in the context of aeon pluralism. But in aeon monism, on the contrary, the question admits a clear answer: the universe had no beginning as it’s a giant cosmic loop. Section 2 briefly introduces conformal cyclic cosmology. Section 3 rebuts an argument provided by Penrose against aeon monism. Section 4 discusses consequences of aeon monism for the philosophy of time by asking whether the model implies a form of eternal recurrence.

## Conformal Cyclic Cosmology

Conformal cyclic cosmology has been introduced and defended in Penrose (2006; 2010; 2014). The view is currently under empirical investigation (Jow and Scott 2020; An et al. 2020) and subjected to further analysis (Meissner and Nurowski 2017; Nurowski 2021; Natarajan and Chandramohan 2021; Bodnia et al. 2023). According to the model, the cosmos is a collection of successive universes: the so-called *aeons*. The beginning and ending of our universe (our aeon) are directly connected to two other, anterior and posterior, aeons. Aeons are represented mathematically in the apparatus of general relativity as relativistic spacetimes, each with an infinite expansion in the future—so, with no big crunches as in other cyclic models.

Central to conformal cyclic cosmology is Penrose’s observation that the past boundary of one spacetime can be connected to the future boundary of another spacetime, at the price of conformal rescaling, namely by stretching the spacetime metric.^2^ A second observation is that only massive particles are sensitive to (non-zero) spacetime distances:

Massless particles [...] do not appear to be particularly concerned with the full *metric* nature of space-time respecting merely its *conformal* (or null-cone) structure. (Penrose 2010,  140)

Thus, the model requires massive particles to disappear, or at least, the rest mass of all particles to go to zero, infinitely far into the future, to allow the aeons to connect. Therefore, an explanation for how massive particles could vanish infinitely far away in the future is required. Penrose offers the following explanation. At the end of the aeon, most of the matter will end up in black holes that can be viewed as converting devices: they transfer energy from the matter fields to the gravitational field, converting material entities into gravitational energy to then release it back again, very slowly, in the form of Hawking radiation mostly constituted of massless particles. Thus, most massive particles will be converted to non-massive particles via black holes. An issue is that not all particles will end up in black holes: some massive particles are likely not to ever end up in one. Penrose thus asserts that we must assume the existence of a hitherto unknown mechanism to explain the decay of all massive particles into massless particles—or, at least, of most of them.^3^

Conformal cyclic cosmology is quite speculative and controversial as it is common with alternative approaches to the standard model of cosmology. However, the approach has the merit of being empirically testable, at least to some degree. Penrose notes that at the end of each aeon, black holes should shrink and disappear, releasing all their energy via Hawking radiation. Before that time, a large number of these black holes will collide and merge. This situation is expected to give rise to two kinds of empirical signatures in the Cosmic Microwave Background (CMB), the remnant light of the big bang. One is associated with black hole merger events that should release energy concentrically in the form of massless particles, leading to concentric signals in the CMB of the next aeon—as massless particles are not bound to the aeons and are expected to cross the ‘Border’ with the subsequent aeon.^4^ A second potentially detectable empirical signature should result from the last bursts of energy emitted in the form of Hawking radiation by super-massive black holes during their final evaporation, generating what Penrose calls *Hawking points* in the CMB. These should be bright spots in the CMB, flanked by concentric rings whose temperature is expected to be lower than the average temperature of the CMB. This last family of concentric rings differs from the first concentric rings associated with the first empirical mark, which do not surround Hawking points and are expected to have a slightly higher temperature than the first class of rings. The claim that significant data favor the conformal cyclic cosmology over the more standard inflation hypothesis is currently under investigation and open to debate (Jow and Scott 2020; An et al. 2020; Lopez et al. 2021; Bodnia et al. 2023).

The proposed approach not only carries empirical consequences but it also provides an explanation for an enigmatic fact that remains otherwise unexplained. Specifically, it provides an explanation for why the entropy is low at the commencement of the universe, a desideratum commonly referred to as the *past hypothesis* (Albert 2000). That the entropy must be low at the beginning of the universe is important to make sense of the second law of thermodynamic that states that entropy can only increase and never decrease. However, the CMB indicates a seemingly excessively high entropy at early times near the Big Bang, which is in direct contradiction with the past hypothesis, posing a significant challenge. As Penrose puts it:

The usual statement of the 2nd Law, that there is a near-universal tendency of the entropy of a system to increase into the future, can be equally be stated in the past direction, namely that there is such a universal tendency to *decrease* as we examine things further and further into the past. Accordingly, the situation at the Big Bang ought to have been one of exceptionally low entropy. This expectation may be contrasted with the most direct observational evidence of the early universe, as provided by the CMB, namely its virtually perfect Planck spectrum [...], which would appear to be indicative of a *maximum* entropy state (Penrose 2018,  1179; emphasis in the original)!

How could we reconcile, then, the past hypothesis with the observed high entropy at the beginning of the universe?

Penrose analyses further the past hypothesis via another, more mathematical, hypothesis: the *Weyl curvature hypothesis*. The latter hypothesis states that the initial maximally low entropy corresponds to the vanishing of the Weyl tensor. Indeed, Penrose (1980) discusses how the gravitational field implies the existence of gravitational degrees of freedom, which means that gravitational entropy increases with the clumping of the elements making up a physical system.^5^ Unlike the entropy attached to the kinematic degrees of freedom of a system, which increases with the diffusion of the system’s elements, gravitational entropy increases with their clumping. As Penrose notes:

The important thing to realize, here, is that with regard to the *gravitational* field, the uniform state is of exceedingly *low* entropy, owing to gravity’s universally attractive nature. In contrast with the behaviour of a gas in a box, for example, where *maximum* entropy would be pictured as something with great spatial uniformity, gravitating bodies, such as systems of stars, would tend to clump more and more in their spatial distribution, as their dynamical time evolution proceeds, representing an increase in the gravitational entropy. The greatest clumping of all occurs with the formation of black holes, which would be accompanied by an absolutely enormous increase in the entropy (Penrose 2018, 1180).

Then, by taking the gravitational entropy as having a much higher value than the kinematic entropy, the resulting global entropy, which is the sum of the kinematic and gravitational entropy, obeys the second law of thermodynamics. The global entropy is smaller at early times than in subsequent cosmic times because the universe starts from a state of low (global) entropy and increases by clumping, which entails the formation of galaxies, clusters of galaxies, black holes, and ultimately, after the slow evaporation of black holes, of massless particles (Penrose 2006,  2760).

The main idea is thus that the existence of gravitational degrees of freedom enables us to understand how entropy can be low in the early universe, despite appearances. Why is this idea called the Weyl curvature hypothesis? The Weyl tensor is a mathematical object that describes the curvature of spacetime that is not due to the distribution of matter and energy. When the Weyl tensor vanishes, it implies that the spacetime is conformally flat, which puts strong constraints on the distribution of matter and energy, resulting in low-entropy states. Thus, the vanishing of the Weyl tensor in a region of spacetime implies that the gravitational degrees of freedom in that region are highly constrained and have a lower entropy compared to regions where the Weyl tensor is non-zero.

Conformal cyclic cosmology provides an explanation for why entropy is so low at the beginning of the universe—in other words, for the ‘extraordinary uniformity’ of the early universe. This is due to the action of black holes that act as suppressors of degrees of freedom, disconnecting them from the later global stages of the universe (Penrose 2014,  885-888). Think about it this way: black holes possess an incredibly high level of entropy, effectively functioning as ‘reservoirs’ for entropy. Consequently, when these reservoirs reach their final stage of evaporation and are destroyed, the total amount of entropy in the universe diminishes drastically. The beginning of the next aeon will inherit the relative uniformity resulting from the final stage of the previous universe, after the total, complete, evaporation of all black holes. This evaporation will have eliminated degrees of freedom, thereby reducing the total entropy of the universe. The resulting low entropy will then pass on to the next aeon. Note that this reasoning is quite speculative as it relies on a number of debatable assumptions, especially regarding black hole physics.^6^

Thus, conformal cyclic cosmology provides an explanation for the low entropy in the early universe. It should be noted, however, that this explanation is not unique and stands in particular in competition with the popular inflation model that traces back the initial homogeneity of the universe to a post-big bang extreme inflation phase.^7^ Yet, providing a coherent explanation for the low initial entropy certainly offers, if not a decisive argument, positive support for the view.

Whether correct or not, conformal cyclic cosmology presents a fascinating picture of the universe that invites philosophical exploration. As a first foray into philosophical discussion, let’s examine whether the preceding and succeeding aeons should be considered as distinct, or as one and the same universe.

## Aeon Pluralism versus Aeon Monism

Penrose explicitly discusses aeon monism by contemplating the possibility of gluing together the two spacelike hypersurfaces $\mathcal {I}^{+}$ and $\mathcal {B}^{-}$ posited respectively at the infinitely far future and beginning of our aeon (Penrose 2010,  147).

However, he then dismisses the idea after conceding that the view would be parsimonious:

The economy of this idea certainly has its appeal, but I think that there could be serious difficulties of consistency which, in my own view, render this suggestion implausible. Basically, such a space-time would contain closed timelike curves whereby causal influences can lead to potential paradoxes, or at least to unpleasant constraints on behaviour. Such paradoxes or constraints do depend upon the possibility of coherent information being able to pass across the $\mathcal {I}^{+}$/$\mathcal {B}^{-}$ hypersurface. Yet [...] this kind of thing is a real possibility in the type of scheme that I am proposing here, and so such closed timelike curves do indeed have the potential to lead to serious inconsistency problems. [...]. For reasons such as this I am not proposing this $\mathcal {I}^{+}$/$\mathcal {B}^{-}$ identification (Penrose 2010, 147).

Here is a reconstruction of the argument:

1. A spacetime temporally closed on itself would feature closed timelike curves.
2. Closed timelike curves lead to paradoxes.
3. A spacetime temporally closed on itself would lead to paradoxes.
4. Therefore, we do not live in a temporally closed spacetime.

The term ‘paradox’ may be taken to refer to a problem of a certain sort. What does it mean that a problem is paradoxical? There is room for disagreement on this question but, under a certain understanding, it’s a situation where *prima facie* unacceptable conclusions follow from *prima facie* acceptable premises. As Sainsbury puts it:

This is what I understand by a paradox: an apparently unacceptable conclusion derived by apparently acceptable reasoning from apparently acceptable premises. Appearances have to deceive since the acceptable cannot lead by acceptable steps to the unacceptable. So, generally, we have a choice: either the conclusion is not really unacceptable, or else the starting point, or the reasoning, has some non-obvious flaw (Sainsbury 2009, 1).

But, then, we should discuss what is the right solution to the problem. Under another definition of the term, a paradox is an unsolvable problem. This is probably the meaning Penrose has in mind so let us investigate whether cosmological closed timelike curves entail unsolvable issues of consistency.

Aeon monism implies that time is closed in a giant cosmic loop (or, more precisely, that timelike trajectories are closed in giant cosmic loops). One might ask, first, whether closed timelike curves, by and large, are problematic and, second, whether the cosmic loop of conformal cyclic cosmology poses distinctive conceptual problems. Let’s start with the first question. As Nerlich (1981) points out, it is useful to compare the question of whether time is closed with the question of whether space is closed. If we consider time only as a dimension, setting aside its direction, flow or relation to causality—in short, all its specific characteristics that distinguish it from space—then the closure of time does not seem especially problematic. Nerlich believes, however, that time is more difficult to close than space because of some of its singular characteristics. Like Penrose (2010,  262),^8^ he links these issues with grandfather paradoxes. Given that the grandfather paradox is the standard way of articulating the central problem with closed timelike curves, let’s have a closer look at it.^9^

David K. Lewis (1976) shows that not all scenarios involving time travel are inconsistent. Although he does not primarily explore scenarios involving closed timelike curves in his paper, his arguments are concerned with showing that time loops do not create logical inconsistencies. His reasoning can easily be applied to the time loops comprising the closed timelike curves.

There are two versions of the grandfather paradox: the *non-modal* and the *modal* versions. The non-modal version exploits an inconsistency in the way the world is. There is an inconsistency between two scenarios: one in which the grandfather was killed, the other in which the grandfather was not killed. Hence, the time travel seems to conjure up a world wherein the grandfather is both killed and not killed. But, as Lewis points out, then what we are describing are *impossible worlds*. The actual world—our world—is of course a possible world and so does not include contradictions of this sort. We should therefore reject these scenarios on the grounds that they represent impossible worlds. If so, they are irrelevant to a discussion of time travel in general, and closed timelike curves in particular.

The modal version focuses instead on the apparent contradiction between the possibility and impossibility of the time traveler killing the grandfather. Lewis’ solution is to analyze these two modal facts with reference to two distinct classes of facts. According to the maximal class of facts, which includes everything existing in spacetime, it is impossible to kill the grandfather, namely to change what is the case somewhere in spacetime. But, according to the class of facts associated with the very moment when the time traveler is ready to pull the trigger, the time traveler can really kill the grandfather.

Thus, two different notions of possibility are defined as two different kinds of compossibility. A fact can be compossible with a class of facts, i.e. *compatible* with one class of facts, while not being compossible with another class of facts. For example, to take Lewis’ example again, a person who has not been taught to speak Finnish *cannot* speak that language in a certain sense (if we include the fact that the person has not been taught to speak Finnish). But in another sense, that same person *can* speak Finnish if we don’t include that fact in the class of facts being assessed, but include the fact that a human being has the ability to speak, in principle, any human language. Likewise for the possibility and the impossibility for the time traveller to kill their grandfather. To be fair, not everyone agrees that the modal problem can be solved in this way. Discussions on these topics are still ongoing, for example on whether certain types of time travel can reintroduce changes in the past (see, for example, Miller 2006; Andreoletti and Torrengo 2019; Baron and Colyvan 2019 and Loewenstein 2022). Despite these reservations about Lewis’ argument, the fact remains that closed timelike curves cannot simply be dismissed as problematic without further discussion.

So closed timelike curves—whether intra- or cross-aeon—are not conceptually incoherent, or at the very least, many of us don’t take them to be problematic. But how plausible is their existence? It is of course difficult to answer such a question. But it is important to remind us that ordinary—meaning, not involving an aeon border—closed timelike curves appear in many models of general relativity.^10^ If one accepts the ability of general relativity not only to describe the real world via a class of models consistent with the empirical data gathered thus far, but also to inform us about what is physically possible, then we should accept that closed timelike curves are physically possible (Earman et al. 2009).

Another question is then whether our confidence in the physical possibility of ordinary closed timelike curves justifies the same confidence in the physical possibility of cross-aeon closed timelike curves. Sure, there is certainly a good deal of mystery about the ontological nature of the boundary between the aeons. But this does not seem to have much effect on what happens in the aeons. Since the cross-aeon closed timelike curves consist of trajectories within aeons and of the crossing of aeon borders, it is difficult to see why the global topological properties of the cross-aeon closed timelike curves, and their physical plausibility, should depend on cross-aeon relations. Perhaps one could provide reasons for why there is a problem here. But, until it is proposed, I see no reason to doubt that ordinary closed timelike curves and cross-aeon timelike curves are both compatible with general relativity, and thus physically possible. So aeon monism, or any hypothesis involving closed timelike curves, cannot simply be dismissed because of the grandfather paradox. This still does not show that aeon monism is an attractive interpretation of conformal cosmology, of course. But it does demonstrate that aeon monism is no less plausible than aeon pluralism.

To end this section, I would like to suggest two positive reasons for giving full consideration to aeon monism. The first reason exploits an analogy between space and time or, more precisely, the spacelike and timelike directions in spacetime. If spacetime is one global entity, then it’s quite natural to deal in the same way with the global topological properties of spacetime in both spacelike and timelike directions.^11^ When it comes to spacelike directions, the two alternative views that a flat space is either infinite, or finite and spatially closed, seem both plausible, and on a par. I believe the same should be true of the timelike directions. The timelike and spacelike directions could be *both* infinite, or both finite and temporally closed, or differ one from each other. But there is no strong reason to expect timelike directions to behave differently from spacelike directions, at least in the framework of conformal cyclic cosmology.

A second reason is that aeon monism should, if not in practice, in principle be empirically falsifiable, and may therefore be more akin to a scientific cosmological model than to a metaphysical interpretation of a scientific model. Suppose that humanity were to become so advanced in the future that it was able to access very large quantities of usable energy. It would then be possible to engineer a super-massive black hole with specific properties, by dragging and merging pre-existing super-massive black holes, calculate the profile of the mark such a super-massive black hole should leave in the CMB of the next aeon, and then carry out an analysis of the CMB of our current universe to see if the two profiles match. The mark would have to be specific enough, to avoid the possibility for it to be produced naturally. This is clearly a science fiction scenario—perhaps a bad one—but it serves well its purpose of underlining that aeon monism could be even more falsifiable than standard conformal cyclic cosmology, if only theoretically, and thus is worth further investigation.

In this section I have dismissed Penrose’s reason for rejecting aeon monism, and given two reasons for giving full credit to the view. To be fair, these reasons are not strong arguments. What I have failed to do is to give a positive, attractive, argument for aeon monism. However, what I have done, is to show that aeon monism is as plausible as aeon pluralism, when it comes to interpreting conformal cyclic cosmology. And, as aeon monism has not yet been studied properly, and since this view is likely to have many repercussions for the metaphysics of time, I propose to start reviewing its main potential implications.

## Eternal Recurrence

One straightforward question, when faced with the ontology of aeon monism, is whether the identity between the past and the future implies a form of eternal recurrence, an infinite repetition of events, as everything is destined to repeat again and again, in a never ending cycle. If so, conformal cyclic cosmology would then provide a scientific background, speculative but scientifically plausible, for the old thesis of the eternal return. Aeon monism seems indeed to imply that what is going to happen will be the same as what happened before, since what has happened and what is going to happen are numerically identical. Aeon monism, in the wake of Heraclitus, the early Stoics and Nietzsche, would thus revive the thesis of the eternal return from its ashes. However, as we will see, aeon monism is inconsistent with the eternal return of things in light of the most plausible metaphysics of time—the* block universe view*.

To see this, consider the set of necessary and sufficient conditions for eternal return. A cosmological scenario of eternal return must satisfy exactly the three following conditions:

1. *Repetition*: everything repeats itself.
2. *Qualitative identity*: everything repeats itself identically.
3. *Infinity*: The number of repetitions is infinite.

The repetition of the same appears to be a prima facie inconsistent notion as how could it be that the distinct things involved in the multiplicity are identical? The tension is resolved by appealing to the distinction between numerical identity and qualitative identity. What eternal recurrence requires is only a succession of qualitatively identical but numerically distinct states or entities.

At this stage, it seems that the sort of closed universe involved in aeon monism substantially differs from the concept of eternal recurrence. To see this, consider Earman’s distinction between eternal recurrence and cyclic time:

It is necessary at the outset to distinguish between two related but different ideas which are sometimes confused: first, the idea that numerically distinct but otherwise similar states occur over and over again in an open time; and second, the idea that the universe progresses through a series of changes only to return to the numerically identical state. Although terminology differs in these matters, I will use *eternal recurrence* to refer to the former, and *circular*, *cyclic*, or *closed time* to refer to the latter (Earman 1995, 203; emphasis in the original).

The distinction between eternal recurrence or eternal return on the one hand, and closed or cyclic time on the other, is quite natural and, I take it, fairly standard (see, e.g., Dowe 2017, 184). Eternal recurrence is usually regarded as conflicting Leibniz’ principle of the identity of the indiscernibles (PII), (see, e.g., Dowe 2017, and references therein on the Stoics). One might see this as a problem or simply a cost of the view. For not everyone takes the PII for granted. Recall that Leibniz substantiates the principle of the PII only indirectly by appealing to the principle of sufficient reason and to theological considerations (Rodriguez-Pereyra 2014). For these reasons and others, there is no agreement in recent discussions regarding the validity of the principle (see, e.g., Hawley 2009).

But note that eternal recurrence does not necessarily run counter to PII if we relax the second *qualitative identity* condition of eternal return. Indeed, this depends on whether the repetitions of a ‘qualitatively identical state’, at the first-order level, can be counted and whether the counting process generates second-order properties associated with the counting and instantiated by the first-order states. Thus, if the *number of iterations* of the first-order state is taken into account in our description of the successive iterations, and considered as a genuine second-order property of the underlying first-order state, this second-order property will act as a distinguishing property. Thus, the physical state considered is not really, *maximally* qualitatively identical to the other physical states that seem indistinguishable (and are indistinguishable at the first-order level). Those are only indistinguishable when we do not include in the domain of comparison the instantiation number that counts the numerically distinct states that look qualitatively identical otherwise. Eternal recurrence only conflicts with the PII if the class of comparison between qualitatively identical states doesn’t include the second-order properties associated with the flow of time, and allowing the counting of successive iterations of cosmic times.

At first glance, it seems that conformal cyclic cosmology implies only a form of cyclic time, not eternal recurrence, just as closed timelike curves in general-relativistic spacetimes appeal to the concept of cyclic time, not eternal recurrence. However, things are more complex than they might seem at first sight, as they depend on several auxiliary assumptions embedded in alternative metaphysical models of (space)time. As we shall see, aeon monism can also be interpreted as implying a form of eternal recurrence if we embrace specific metaphysical views.

Consider the block universe view, the most popular approach in the philosophy of time, here defined as an eternalist B-theory of time: past, present and future entities exist *simpliciter* in a four-dimensional spacetime (see e.g. Smart 1963; Mellor 1998 and Sider 2001). Eternalism states that what we describe as past, present and future entities, equally co-exist. Coupled to the B-theory of time, those notions of past, present and future are re-framed in terms of relations of anteriority, simultaneity and posteriority between events or objects. Relativistic physics further demands that we conceive of the world in terms of spatiotemporal relations instead of spatial and temporal relations. The B-theory of time then asserts that the ontology of time is exhausted by those relations, and there is no need to add dynamic A-properties, or tensed facts, in the fundamental building blocks of the natural world. The block universe view, when coupled with aeon monism, requires a final conceptual maneuver. Indeed, we can refer to two very different things as ‘time’ in the resulting approach. One of these things is the set of directions between timelike separated events in the four-dimensional manifold, the other is the directed connection between the two ‘temporal sides’ of the aeon. One must thus stipulate that these two things partake of the same, broader, phenomenon: time.

The block universe interpretation of aeon monism implies that there is *no repetition*, as all things happen exactly once: the totality of reality is exhausted by a single universe—a unique spacetime—temporally closed on itself. The notion of repetition requires a notion of multiplicity. Provided that there is only one universe, there can be no repetition. As the concept of repetition is involved in the three conditions of eternal recurrence, the block universe interpretation is inconsistent with it. The block universe interpretation thus depicts a cyclic time, but not eternal recurrence. Note that by the same reasoning the block universe view, independently of aeon monism, also implies rejecting repetition in the context of general-relativistic solutions involving closed timelike curves. Things happen exactly once on each closed timelike curve.

Although the block universe view is fairly standard in philosophy of physics (see e.g. Wüthrich 2013; Smeenk 2013; Callender 2017; Le Bihan 2020), and popular in the metaphysics of time, others, especially in the metaphysics literature, defend alternative views. Three influential alternative views to the block universe view are: the *growing block theory*, the *moving spotlight theory* and *presentism*. All three global views share a commitment to the A-theory of time, which asserts that things really do flow in time, in a way that is not purely metaphorical—for instance, by instantiating successively transitory properties, or due to the existence of primitive tenses.^12^ In what follows, I will examine the implications of these three metaphysics of time when combined with aeon monism. Readers committed to the block universe theory might perhaps contend that these A-theories are irreconcilable with contemporary physics and thus do not really merit such a discussion. However, in the context of this article, I wish to remain as agnostic as possible about which metaphysics of time is best, setting aside my own commitment to the block universe view. In the spirit of calling on metaphysicians of time to consider conformal cyclic cosmology in more detail, the aim of the discussion is to provide a catalog of the implications of aeon monism for the philosophy of time, more generally, in its diversity of approaches.

The growing block theory combines a dynamical view with no-futurism, the claim that past and present entities exist, in contrast to future ones. The view, defended for instance by Tooley (2000) faces a now-now objection (Braddon-Mitchell 2004) and is incompatible with general relativity, at least if we take seriously its whole space of solutions, as some of them include closed timelike curves (Le Bihan 2014). The view is also logically incompatible with aeon monism for the same reason, as it requires the numerical identity of the non-existent future aeon with the existing past aeon. It is hard to make sense of a situation in which a class of entities could both exist when considered in one set of directions (in the past timelike directions of spacetime) and fail to exist when considered in another set of directions (in the future timelike directions). Thus, the growing block theory is simply incompatible with aeon monism.

The second view, the moving spotlight theory, famously criticized by McTaggart (1908), has recently seen a surge of interest among metaphysicians (see, e.g., Skow 2009; Cameron 2015; Deasy 2015; Miller 2017; 2019; Spolaore and Torrengo 2021). The moving spotlight approach, combines eternalism with an A-theory of time. A similar yet distinct view is the *wave theory of time* (Effingham 2023), where the transitory feature of the present is the constitution of objects by hunks of matter, a view which is not eternalist if eternalism is defined in reference to the existence of ordinary objects, rather than other entities (such as four-dimensional parts of spacetime, or chunks of matter). As both theories are structural-ly similar in their requirement of a present moving within spacetime, I will focus on the moving spotlight theory, on the understanding that the lessons learned along the way also apply to the wave theory.

The moving spotlight theory relies on the view that the flow of time is a *transitory monadic property* that applies successively to different sets of events, thereby defining a global separation between three domains of reality: the past, the present and the future. Call the view *standard realism* about the flow of time.^13^ Standard realism about the flow of time is usually seen as standing in conflict with special and general relativity, and for this reason remains a minority view in the contemporary philosophy of time (Saunders 2002). Coupled to aeon monism, we end up with an exotic sort of moving spotlight theory according to which the flow endlessly sweeps out the four-dimensional spacetime, temporally closed on itself. This entails a form of eternal recurrence via the repetition of the instantiation of the monadic property of being present that transits endlessly in spacetime. Such a view leads to a slight departure from the historical theory of eternal return though, as what is repeating are not things themselves, but the application of the transitory properties of being past, present and future, to physical systems.

Finally, a third alternative ontology of time is presentism, the view that only present entities exist.^14^ Does the combination of aeon monism and presentism entail eternal return? The situation appears at first sight to be quite similar to the moving spotlight theory, except that in this case we do not observe the migration of transitory A-properties in spacetime but the successive configurations of qualitatively identical states. However, the combination of presentism and aeon monism proves difficult to analyse. Usually, in standard open-time scenarios of eternal recurrence, recurrent states are regarded as qualitatively identical but numerically distinct. But are they also numerically distinct in the closed time hypothesis? One reason to doubt it is that in aeon monism, there is only one iteration of the universe. To appreciate this point, consider an alternative possibility: it could have been the case that the cosmic loop was formed not by a single universe temporally closed on itself, but by two, three or any other number of universes, thus forming a closed chain of a certain length. That there is no non-trivial metric other than ‘one’ to measure the number of iterations of the universe casts doubt about whether two successive iterations of the same qualitatively identical state of the universe (in ‘two’ successive aeons) are really numerically distinct. After all, presentist aeon monists must claim that there is only one iteration of the universe, although this single iteration of the universe is done only at the rhythm of ‘one time at a time’. If this is indeed the correct presentist analysis of aeon monism, then there is no true repetition, and no eternal recurrence—there is only the *succession* of numerically identical states. The very same state would be both before and after itself (just as something could be on the right of itself, in a closed space). But this analysis is only one possibility, the other being that it is possible to count successive, numerically distinct, iterations of the states in the loop.

Whether the presentist ontology leads to eternal recurrence will thus depend on whether the successive iterations of the states can be counted.^15^ Is that so? Answering this question is not trivial. Unlike the moving spotlight theory, the presentist does not have such easy access to an ontology that allows them to anchor truths about the past, and the future, and to count the successive exemplifications of the numerically distinct physical states. Now, this appears to be just one particular instance of the truthmaking objection raised against the presentist (Bigelow 1996; Keller 2004; Asay and Baron 2014; Baron 2015). If they can ground truths about the past, perhaps they could also ground truths about the number of past iterations of the state, and future iterations of the state to come. In sum, this discussion shows that the question of whether the presentist interpretation of aeon monism leads to eternal recurrence depends on how to ground (or not to ground) facts about the number of successive iterations of the universe at an instant.

## Conclusion

Conformal cyclic cosmology does not by itself privilege aeon pluralism over aeon monism, as no compelling reason has been offered against aeon monism so far. While speculative, aeon monism is such a startling cosmological view that it is worth exploring its philosophical repercussions. Aeon monism is an interesting and relatively simple case study, which allows for the study of the fate of various metaphysics of time in cyclical cosmological models more generally, highlighting the problems associated with the very idea of cosmic recurrence.

A first preliminary result, assuming that the block universe view is the correct ontology of time, is that aeon monism is incompatible with eternal recurrence. Only rival and dynamic views of the nature of time—such as the moving spotlight theory and presentism—could provide the resources necessary for the development of eternal recurrence within the framework of aeon monism.

Overall, conformal cyclic cosmology might yield profound consequences for our understanding of the (lack of) genesis of the universe, with many philosophical implications. Thus, both conformal cyclic cosmology and aeon monism could benefit from a greater involvement of philosophers to further elucidate their conceptual intricacies and philosophical ramifications.

## Footnotes

1 On eternal inflation and brane cosmology, see respectively Freivogel et al. (2006), Guth (2007) and Nomura (2012), Guth and Nomura (2012), Carroll (2019) and Arkani-Hamed et al. (1998), Shifman (2010). On loop quantum cosmology, see for instance Bojowald and Brahma (2018); Brahma (2020), and, for philosophical discussion, Huggett and Wüthrich (2018).

2 The rescaling is done by adding a spacelike hypersurface to the past boundary of spacetime. The conformal geometry of spacetime can be extended beyond the boundary by infinitely stretching the metric at the Big Bang singularity, the conformal factor becoming infinite there. Similarly, for the cosmic future, the geometry can be modified by adding a spacelike hypersurface at the future timelike infinity in the conformal completion. There, the conformal factor goes to zero, corresponding to the squashing of the metric. See (Penrose 2006,  2761).

3 Penrose nuanced his claim that *all* massive particles should disappear in his Nobel Prize lecture (Penrose 2020, 26:30). This seems to be one of the most serious issues with conformal cyclic cosmology.

4 Search for such an empirical signature associated with conformal cyclic cosmology and ruling out inflationary cosmology remains inconclusive at the moment as “[the low-variance circles of Gurzadyan and Penrose (2010)] can naturally occur in a Gaussian CMB sky consistent with the predictions of the inflationary cosmology” (Hajian 2011, 2 [on the ArXiv version: https://arxiv.org/pdf/1012.1656]).

5 Some disputes the coherence of the concept of gravitational entropy, see, e.g., Callender (2010) and references therein.

6 For example, Penrose assumes that black holes are genuine thermodynamic systems, an assumption that is not universally accepted (see Dougherty and Callender 2016; Chua 2023 for criticism, and Wallace 2019 for a defense of the claim), and that non-unitary physical processes are acceptable. The predominant view among black holes physicists is that unitarity should be protected at all cost. However, a number of physicists and philosophers of physics, especially from the relativistic community, accustomed to solutions of general relativity that are not foliable in Cauchy surfaces, find it easier to accept the existence of non-unitary physical processes (see, e.g., Belot et al. 1999).

7 In some sense, conformal cyclic cosmology is also an inflation model, but one with an inflation that took place in a previous aeon: “in [conformal cyclic cosmology], the role of inflation is taken over by the exponentially expanding ultimate history of the previous aeon. In a sense, ‘inflation’ of a sort does take place in [conformal cyclic cosmology], but it occurs ‘before’, rather than after the Big Bang (echoing an earlier idea expressed by Gasperini and Veneziano 2003)” (Penrose 2018,  1186).

8 “An example of such an inconsistency problem is the so-called grandfather paradox in which a man travels back in time and kills his biological grandfather before the latter met the traveller’s grandmother. As a result, one of the traveller’s parents (and by extension the traveller himself) would never have been conceived. This would imply that he could not have travelled back in time after all, which means the grandfather would still be alive, and the traveller would have been conceived allowing him to travel back in time and kill his grandfather. Thus each possibility seems to imply its own negation, a type of logical paradox” (Penrose 2010,  262).

9 Another question concerning the possibility of closed timelike curves in general, which will not be examined in this work, is whether they contradict the irreversibility of certain physical processes Rovelli 2019. For a negative answer to this question, see Nikitenko (2021) and Doboszewski (2022, Section 5.6).

10 Gödel (1949) offered first examples of such solutions in a special issue of *Reviews of Modern Physics* dedicated to Einstein’s seventies birthday; see Smeenk and Wüthrich (2011) for an overall presentation of modern solutions.

11 We ignore here the timelike directions at the points in the manifold associated to trajectories that end up in black holes, to focus on timelike, null and spacelike directions at all the other points.

12 Dowe (2017) offers an interesting comparison of A- and B-theories of time in the context of closed time, relevant to this discussion, and argues that the A-theory should also be considered as a serious candidate for making sense of closed time.

13 It would be interesting to examine other alternative A-theories in future work, such as Fine’s non-standard A-theories (Fine 2005; 2006, see Savitt 2016 for a discussion).

14 See, e.g., Bigelow (1996); Bourne (2006); Crisp (2007); Tallant and Ingram (2015); Hinchliff (2000).

15 A third option would be to say that there is eternal repetition and recurrence in a way that cannot be counted. Perhaps this could be achieved by attributing a determinable property without a determinable value to the recurring states. But as this is a long shot, I won’t discuss it further here.
